# Adaptation and Validation of the Food Neophobia Scale: The Case of Hungary

**DOI:** 10.3390/foods10081766

**Published:** 2021-07-30

**Authors:** Zoltán Szakály, Bence Kovács, Mihály Soós, Marietta Kiss, Nikolett Balsa-Budai

**Affiliations:** Institute of Marketing and Commerce, Faculty of Economics and Business, University of Debrecen, Böszörményi Str. 138., H-4032 Debrecen, Hungary; szakaly.zoltan@econ.unideb.hu (Z.S.); kovacs.bence@econ.unideb.hu (B.K.); soos.mihaly@econ.unideb.hu (M.S.); budai.nikolett@econ.unideb.hu (N.B.-B.)

**Keywords:** food neophobia scale, FNS, validation, factor analysis, consumer segmentation, Hungary

## Abstract

Food neophobia is the fear or loathing of novel food, which may result in the rejection of the unfamiliar food item. The most frequently used and most reliable tool to measure adult food neophobia is the Food Neophobia Scale (FNS), which has been translated into several languages, making it possible to compare food neophobia levels around the world. The main objective of this research has been the adaptation and validation of the FNS in Hungary. In order to achieve the research objectives, a questionnaire survey was conducted on a representative sample of 500 adults; and, primarily, multivariate statistical tools were used. We found that despite the Hungarian population’s strong neophobic tendency, neophobia and neophilia are present at the same time. We identified two distinctive factors in the course of the exploratory factor analysis (“Willingness and trust” and “Rejection and particularity”), which distinctly separate the negatively and positively worded (reversed) FNS items. Based on these factors, four clusters were identified. Those belonging in the group of adventurous open-minded individuals constitute an ideal target group for the manufacturers of novel food items as well as products with unusual flavors, especially if those products also have health-enhancing and eco-friendly qualities.

## 1. Introduction

Food neophobia is the fear or loathing (perhaps even disgust) of novel food, which may result in the rejection of the unfamiliar food item. This, in part, is rooted in evolution, but traditions also influence which food items become accepted by the individual. Every single food item represents a possibility and a risk at the same time [1], in that there is a possibility to ingest different nutrients, but it can turn out to be dangerous as well. However, to be able to take advantage of being an omnivore and being able to gain nutrients from several sources, the individual must be willing to sample (accept) new food items as well. According to Rozin [2], this is the omnivore’s dilemma, that is, avoidance and rejection, as well as the acceptance (sampling) of new food items that are present at the same time.

The opposite of neophobia is neophilia; representatives of this group will sample anything and their food choices are primarily generated by curiosity. The only difference between the sampling willingness of neophobic and neophilic people can be detected in the case of novel food items, not in the case of food items that are already familiar to them [3]. It is important to note that neophobic consumers not only show less willingness to try new foods but they will also find these food items less tasty after sampling than their non-neophobic peers [4,5,6]. Therefore, neophobia has an effect on the quality and variety of eating as well [7,8], with the result that neophobic individuals may have a less diverse and more monotonous diet than their neophilic peers [9].

Cultural diversity and globalization have made it possible for novel food items to appear on the market, besides traditional foods. Given that the Regulation on Novel Food [10] recently came into force, we can expect even more new and innovative food items to appear on the shelves of grocery stores, thus, the study of neophobia has become a topical issue once again [4,11,12,13]. This is all the more so given that, even though food items are becoming ever safer due to state-of-the-art technologies used in their production, the fear of unknown food items is still an existing phenomenon [14,15].

### Theoretical Background

Several social, psychological and biological factors define whether an individual will accept or reject new food items of unknown origin [16].

First of all, familiar descriptors of food can lower neophobia, but it is important to clarify what we mean by the notion of “familiarity.” From the perspective of neophobia, an unknown product can be an entirely novel product, but it can also be an already known type of product with new ingredients, flavors, or, conversely, familiar flavors used in an entirely new product. A new combination of already familiar flavors also counts as novelty [5]. Familiarity in the cultural sense—for instance, a food item that is traditional in the given country but unknown to the individual—does not decrease neophobia, because personal experience is always necessary to decrease it [17].

Culture can also influence neophobia [12,18,19]. Those who travel more, and those who are more open to the culture of other countries, tend to show less neophobic behavior [9,18,19]. In addition, higher levels of neophobia positively correlate with a lack of visits to restaurants offering different ethnic foods [9,20]. Furthermore, the willingness to accept different ethnic food items can be influenced by the notions the individual has about the given culture [12]. For neophobic people, the origin of a food item is more important than for their neophilic peers [21].

When it comes to personal characteristics, food neophobia positively correlates with the tendency towards anxiety and neurotic behavior [22], whereas openness and extraversion negatively correlate with neophobia (especially in men) [6]. Moreover, neophobia can also be caused by a fear of gaining weight [23].

The connection between neophobia and gender is not entirely clear. Some research results [17,21,24] indicate that neophobia can appear in a stronger form in men than in women; surveys conducted by Knaapila et al. [6] and Sogari et al. [25], however, came to the opposite conclusion, although others did not discover gender-based differences [4,7,13,18,23,26].

Concerning other background variables, some research found a negative correlation between neophobia and socio-economic status, cultural diversity [20,21,27] and level of education [17,18,21,28,29]. Furthermore, neophobia also shows a negative relationship with the extent of urbanization. This can probably be explained by the phenomenon that urban dwellers are typically more familiar with more types of food (thanks, for example, to the ethnic restaurants found in cities) [17,21,27,29].

Besides this, acceptance of novel food also depends on age. Neophobia typically appears in its strongest form between the ages of 2 and 6 years [26,30], followed by a declining tendency with time, then stabilizes by young adulthood, and later on declines again [4,22,31]. This is supposedly due to the fact that the individual will gather more experience with age, therefore fewer and fewer food items will remain unfamiliar [9]. Interestingly, though, neophobia can increase again with the advent of old age as the senses become duller, which will make identifying and distinguishing flavors harder. A further reason for neophobia in older adults is that, due to the effects of globalization, ethnic foods have appeared only recently, but they tend to distrust these food items [4,9,17,18,22].

The emergence of neophobia can be rooted in genetic and environmental factors as well [6,32,33,34]. The three types of food most frequently causing neophobia are vegetables, meat and fruits [9], and this phenomenon may have evolutionary reasons, because these are the food items most commonly associated with toxins, bacteria and allergic reactions [9,26].

When it comes to the development of food neophobia, it is key which flavors became accepted during a person’s childhood, because introducing a diverse diet early negatively correlates with neophobia [8,31,34]. In order to avoid the consumption of poisonous plants, children naturally object to tasting bitter-tasting food items (primarily vegetables and fruits) when they have no previous experience with the foods in question [22,26,30]. A bitter taste may, in time, be accepted, but repeated sampling is a prerequisite for this [9,35]; in the case of neophobic adults, however, the person remains sensitive to tasting bitter flavors [32]. Furthermore, children can also independently develop schemes as to what they are willing to accept and how, which can be influenced by the color or smell of the food on offer, but even by the distance between food items on the plate [22].

Parents have a pivotal role in the development of childhood neophobia. Those children who previously had more negative experiences when trying new food items (for example, parental force-feeding) will typically experience stronger cases of neophobia than their neophilic peers [22,34]. Moreover, parents rarely offer their children food items that they themselves are unfamiliar with, or that they do not like [16]. As a consequence, these children will experience a higher level of neophobia than their peers with non-neophobic parents [16,23,26,33,34].

On the other hand, neophobia in childhood and adulthood can be decreased by positive information about the new food item, and willingness to sample and accept a food item is increased if a new food is accompanied by familiar flavors, if the new food item resembles an already familiar food, or if it appears as part of similar food combinations [8,9,16,25]. Continuous and repeated positive experiences—usually 10–15 positive experiences are necessary for a new food item to be integrated into a person’s diet—reduce the objection to accepting the food item and, as a result, also reduce neophobia [9,16,17,26,30,31,32,35]. Moreover, social factors can also contribute to the decrease of neophobia; the more people eat together, the greater the willingness to try out a new food item and the lower the neophobia [22,30].

Neophobia can also be part of—albeit not the sole reason for—picky eating habits [9,22]. Although the notions of neophobia and picky eating habits are often blended together [33]—in both cases, we can speak of the rejection of food [31]—they are, in fact, not the same phenomenon. Whereas in the case of neophobia, the person only rejects new/unfamiliar food items, in the case of picky eating habits a majority of food items are rejected, which includes both unfamiliar and already familiar ones [22,31,33]. Furthermore, picky eating habits include the consumption of an insufficient amount of food as well as the rejection of certain food items based on their texture [31].

The most frequently used and most reliable tool to measure adult neophobia is the Food Neophobia Scale (FNS) [9] developed by Pliner and Hobden [36]. The scale consists of 10 items, 5 of which refer to neophobic behavior, and the other 5 to neophilic behavior. The higher the FNS value, the more strongly it indicates neophobia. The FNS can be an appropriate method to measure the willingness to sample new food items, to study the acceptance of exotic cuisines and to explore expectations towards novel food items [11].

Over the years, the original FNS has been translated into several languages, including Swedish [16], Finnish [17], French [8], Korean [12], (European) Portuguese [28], (Brazilian) Portuguese [29], German [21,23], Dutch [37], Italian [18] and Chinese [13]; but few or no studies have been conducted in Eastern Europe [38]. Translated and validated scales adapted to various national markets make it possible to compare nations based on their FNS values [19,21]. However, Ritchey et al. [39] called attention to the phenomenon that, due to cultural differences, after the scales have been translated, certain statements may become incomprehensible to a given nation, which can distort the results.

Based on the literature, our research objectives were as follows:

- Identifying a factor structure of food neophobia in Hungary, based on the items in the Food Neophobia Scale (FNS) questionnaire;
- Examining the applicability of the FNS measuring tool to the entire Hungarian adult population;
- Identifying consumer segments (clusters) in Hungary based on the extent of food neophobia.

## 2. Materials and Methods

### 2.1. Sampling Method

Data collection was carried out in October 2020 in Hungary by means of personal interviews. The study obtained ethical approval on 15 September 2020 (approval number: GTKDH/48/2021) from the Research Ethics Committee at the University of Debrecen, Faculty of Economics and Business. Participants gave informed consent before taking part in the research. The national questionnaire-based survey was representative of the population in terms of gender (χ^2^(1) = 0.803; *p* = 0.370) and age group (χ^2^(2) = 0.3.901; *p* = 0.142). In the sampling process, representativeness was also ensured for regions (χ^2^(2) = 0.187; *p* = 0.911) and settlement types (χ^2^(2) = 7.279; *p* = 0.064) (quota sampling). In the assigned settlements, each interviewer was given a randomly selected starting address. In ascending order by house number, the interviewers then began the questioning at the third house on the same side of the street, and then, if they were done there, they continued at the next third house. During the compilation of the sampling plan, it was also ensured that the interviewers should have no problem whether they were conducting the questioning in a district with detached houses or in a district with blocks of flats. From among the residents of the households visited, those participants whose birthday was the closest to the date of the survey were selected for the interview. With this method, randomness was ensured only in each stratum. The sample consisted of 500 persons. Given that in Hungary the number of adults is approximately 8 million [40], and with a 95% confidence level and a 5% margin of error (based on [41]), the required sample size is 385 respondents. Consequently, the sample size was appropriate for reaching the research objectives. Table 1 shows the percentage distribution of the socio-demographic groups of the individuals involved in the survey and the population composition according to the previously mentioned four factors.

### 2.2. Structure of the Questionnaire

The questionnaire was made up of two parts: the 10 items of the FNS and 7 questions concerning socio-demographics (see Table 1). In the questionnaire, we used the 10 items of the original FNS developed by Pliner and Hobden [36], translated into Hungarian with the back translation method. The questionnaire was finalized after conducting a pilot survey among 20 Hungarian adults, which resulted in a minor change in item 5 and item 10 (similarly to some previous findings [43], the terms “ethnic” food and restaurant were substituted because in Hungarian, the terms “foreign” food and restaurant are more commonly used and better understood). Respondents had to answer each statement on a Likert scale of 1–7, where 1 stood for “strongly disagree,” while 7 stood for “strongly agree.” Similarly to some previous research studies [12,17,18,19,29], based on their FNS total scores, respondents were separated into three categories, where cut-off points were the FNS total scores ± standard deviation; i.e., if the respondent’s FNS value was lower than the total score minus standard deviation, then he/she was sorted to the neophile group; if the FNS value was higher than the total score minus standard deviation but lower than the total score plus standard deviation, then the respondent belonged to the group of neutrals; and if the FNS value was higher than the total score plus standard deviation, he/she was classified as neophobic. We examined the effect of demographic background variables on food neophobia with the independent-samples t test (in the case of gender) and with ANOVA (in the case of the other variables).

### 2.3. Data Analysis

First, exploratory factor analysis (EFA) was performed on the model with the aim of exploring whether the pre-hypothesized factor structure appeared in our sample and whether we were able to measure the desired attitudes (factors that can be defined as latent variables). Then, we examined the reliability of the scales within the measurement model of the revealed latent variables using the Cronbach’s alpha index and the composite reliability index and omega. This was followed by a confirmatory factor analysis (CFA), the purpose of which was to prove the convergent validity, i.e., whether our empirical model fits the assumed model. Discriminant validity—which tests whether concepts or measurements that are not supposed to be related are actually unrelated—was examined according to the Fornell–Larcker criterion [44]; i.e., the square root of both factors’ AVE (average variance extracted) value should be higher than the correlation coefficients between the latent variables. For further examination, data reduction by principal component analysis was performed separately on the latent variables in order to obtain latent variables free of cross-loadings. The segmentation was performed by cluster analysis, which consisted of two main steps: first, the number of clusters/segments was determined by hierarchical cluster analysis, and then the cluster analysis was carried out using the K-means method, in which the cluster means were determined by the applied statistical software. In order to examine the clusters, cross-tabulation analysis and simple hypothesis tests were used. For CFA, v3.5.0. of R Statistics in the RStudio editor was used (The R Foundation, Vienna, Austria), and all additional tests were performed in v23.0. of IBM SPSS Statistics (Armonk, NY, USA).

## 3. Results

### 3.1. Exploring the Food Neophobia Scale

Table 2 illustrates descriptive statistics relevant to each item on the FNS. The mean value of the total FNS is 39.75; using the value of the ± standard deviation as the cut-off point, we can say that the majority of respondents (65.8%) belong in the neutral group, whereas the proportions of the neophile and neophobe groups are almost the same (17.4% and 16.8%, respectively). These results draw attention to the fact that fear of the new and seeking out novelties coexist in respondents. This is indicated by the high values of standard deviation and coefficient variation as well.

Respondents mostly do not like going to ethnic restaurants (mean: 4.70), nor do they like sampling food items where they do not know what it contains (mean: 4.52). The skewness of the sample distribution also supports these results in the case of both items. A majority of respondents agree that they do not constantly sample new and different foods (mean: 4.14), and they typically do not sample new foods at gatherings and parties (mean: 4.08). Consumers are, however, less afraid of foods which they have never had before (mean: 3.68), and they (typically) trust new foods (mean: 3.61). They are also in agreement with the statement that they eat almost everything (mean: 3.18).

If we examine demographic background variables (Table 3), we find that there is no significant difference in FNS values when it comes to gender and settlement type. However, food neophobia significantly grows with age (*p* < 0.001); while in the 18–39 age group the mean FNS value is 36.87, in the 40–59 age group it is 39.02 and in the 60 or older age group it is 43.47. There is a significant relationship between the FNS value and the level of education (*p* < 0.001)—people with higher qualifications have lower FNS values, thus they can be seen as less neophobic than people with lower levels of education (the mean value for university graduates is 38.67, whereas for those who only finished primary school it is 45.19). Finally, FNS values significantly vary according to subjective income as well (*p* < 0.001), because those in a better financial position are less neophobic than people in a worse financial position (the mean value for the highest income bracket is 36.2, whereas for the lowest income bracket is 46.76).

### 3.2. Results of the Exploratory Factor Analysis

The results of the exploratory factor analysis can be seen in Table 4. EFA resulted in a model with two factors. The first, strongest factor is “Willingness and trust”, which explains 47.927% of the variance. The higher factor weights indicate that the dimension influences Hungarian consumers’ thinking to a great extent and it is distinctly separated from the second factor, i.e., “Rejection and particularity”, which includes aversion towards new food items and picky behavior and explains 17.325% of the variance. We deleted item 9, because of its low explanatory power.

### 3.3. Examination of the Applicability of the Model

Based on Cronbach’s alpha, the composite reliability indicator and McDonald’s omega (Table 5), our applied scales can be regarded as reliable, and reliability cannot be further increased by the removal of items [45]. Omega assumes a congeneric model, which means that factor loadings are allowed to vary in a CFA model.

In CFA, the measurement and structural parts of the model (the hierarchical relationship between measurement and latent variables) were developed according to EFA, supporting our preliminary assumptions. According to the CFA indicators (Table 6), the internal validity of the model can be accepted and the CFA indicates a good fit, so the empirical model can fit the theoretical model. In the CFA, the only concession we made was to allow the covariance between the measurement variables for a given latent variable in the calculations in all the cases where the modification index showed outliers.

The examination of discriminant validity resulted in that the square root of both factors’ AVE value is higher than the correlation, therefore, the Fornell–Larcker criterion is satisfied (Table 7).

### 3.4. Segmentation Based on the FNS

In the course of the segmentation of the sample, the segment-forming criteria were the factors previously established based on the FNS. Given that our data proved to be suitable for segmentation, we used hierarchical cluster analysis to identify the number of clusters, and we also examined if there were any outliers. As we did not find any outliers and we established the number of segments as 4 clusters, we ran the cluster analysis with K-means clustering. The clusters thus formed differ significantly (*p* < 0.001) in variance analysis, therefore the result of the segmentation is valid. Out of a sample of 500, we managed to fit 476 (95.2%) respondents into 4 clusters, namely, “Careful and picky open-minded”, “Non-picky older adults”, “Traditional rejectors” and “Adventurous, open-minded”. In the following, each cluster is described in detail (Table 8).

Cluster 1—Careful and picky open-minded. This group constitutes 21.4% (102 individuals) of the sample. The ratio of men and women is balanced in the cluster, and the age group 18–39 is the largest one, while the proportion of those from the 40–59 age group is less than the average. The size of the group increases with the level of education. Singles are highly overrepresented, whereas widows/widowers and divorced people are underrepresented. Members of this cluster are typically in a better financial position; the two highest income levels are represented in proportions significantly higher than those in the sample. Very health-aware consumers strongly dominate the segment, and the same is true for environment-awareness. Members of this cluster typically live in towns; those living in other types of settlements are highly underrepresented.

Members of this cluster are demanding and they are careful with novelties. Out of all the segments, they are the most characterized by pickiness in their behavior. If they do not know what a food item contains, they do not sample it. Despite this, like the members of Cluster 4, they visit new ethnic restaurants relatively frequently, and they are also willing to sample unfamiliar food items at social gatherings, but, at the same time, they are careful and circumspect when it comes to sampling new, unfamiliar food items. From this perspective, they present ambiguous behavior—on the one hand, they are careful and selective, while, on the other hand, they are open-minded and curious.

Cluster 2—Non-picky older adults. This is the smallest cluster out of the four segments (17.7%, 84 individuals). Women are somewhat over- and men underrepresented, and the number of respondents belonging in this cluster increases with increasing age (12.9% of 18–39 and 25% of 60+ year-olds). Within the group, those who only finished primary and vocational school dominate. Widows and widowers are represented in an outstandingly high proportion, whereas people in other groups based on marital status are underrepresented. The group is characterized by average financial means; none of the subjective income levels are dominant in the cluster. They are also average when it comes to being health-aware and environment-aware. Typically, members of this cluster live in smaller settlements; those living in smaller municipalities are strongly overrepresented.

The members of this cluster are only slightly picky, so they can be regarded, in this respect, as “not demanding.” We can suppose that they do not visit new, ethnic restaurants due to financial reasons, despite this, they are not afraid of novelties, they are willing to try ethnic foods which they encounter for the first time or which they have not sampled before. Like Cluster 4, but unlike the other clusters, they tend to trust new food items. Unlike Cluster 3, they tend to consume all kinds of food items. Their behavior is likely defined by their financial circumstances and mainly by their level of education.

Cluster 3—Traditional rejectors. This cluster constitutes 29.4% (140 individuals) of the respondents. Men are overrepresented in the segment, as well as people in the 60 years or older age group, whereas women and people in the 18–39 age group are underrepresented. When it comes to the level of education, the dominant groups are those who finished only primary school or vocational school. Marital statuses are relatively balanced in the cluster; divorced, widowed and married people are represented in slightly higher than average proportions. Members of this group typically live in worse than average financial conditions. They are moderately health-aware and environment-aware; typically, they cannot be regarded as conscious consumers. Those living in the capital are overrepresented.

Their behavior towards novel food is typically characterized by mistrust and rejection, out of all the clusters theirs is the one most strongly characterized by this attitude. An indication of this tendency is that they are especially afraid of foods they have not sampled before, but they also dislike foods originating from other countries.

Cluster 4—Adventurous, open-minded. This cluster is the most populous out of the four (31.5%, 150 individuals). Men are slightly underrepresented in the cluster, and the 18–39 and 40–59 age groups are highly overrepresented. The cluster is dominated by individuals who finished high school and university, whereas those with primary and vocational school education are underrepresented. Members of this cluster typically live in balanced financial circumstances, and the ratio of mostly health-aware and mostly environment-aware consumers is the highest in this segment, out of all the groups. The division of settlement types is balanced in the cluster.

Members of this cluster can be seen as the most open towards novel food items; they trust foods they have not sampled before, and they are the most willing to try ethnic foods they have not seen before. Similarly to Cluster 1, they visit new ethnic restaurants relatively frequently, seek out new flavors and novelties, are adventurous, and therefore, are the least characterized by a preference for familiar tastes.

## 4. Discussion

The Hungarian adult population’s total FNS mean value is 39.75 (standard deviation (SD) = 12.599). This mean value is relatively high in international comparison; in the case of a representative sample of the Finnish adult population, their FNS value is 38.0 (SD = 10.5) [20], while according to another research study [17] it is 33.9 (SD = 114), for Spanish adults it is 31.74 (SD = 10.98) [4], for Swedish mothers with children it is 25.0 (SD = 7.5), for fathers is it 27.0 (SD = 9.1) [16], for Korean adults aged 20–40 it is 33.5 (SD = 9.0) [12], for Canadian university students it is 34.51 (SD = 11.86) [36], for university students in the USA and Lebanon it is 29.8 (SD = 11.7) and 36.4 (SD = 9.8), respectively [19], for Brazilian university students it is 27.5 (SD = 11.1) [29] and for Chinese college students it is 33.59 (SD = 8.14) [13].

Nearly two-thirds (65.8%) of the Hungarian sample can be regarded as neutral from the perspective of food neophobia, while the ratios of neophilic and neophobic respondents are almost the same (17.4% and 16.8%, respectively). These ratios are almost identical to those found by D’Antuono and Bignami [18] in Italy (neophile: 17%, neutral: 66%, neophobe 17%) and with ratios measured by Tuorila et al. [17] in Finland (17%, 66%, 17%, respectively), however, they significantly differ from the ratios found by Choe and Cho [12] (15.6%, 71.4%, 13%, respectively), which suggests that Korean respondents have less extreme attitudes towards novel foods than Hungarians. Among Brazilian consumers [29] there is a lower proportion of neophiles (10%) and a higher proportion of neutrals (72.5%) than among Hungarians, just as in the Lebanese survey conducted by Olabi et al. [19] (7.9% and 70.6%, respectively), whereas according to the former survey, the ratio of neophobes was nearly identical to the Hungarian ratio (17.5%), while in the latter it exceeded the Hungarian ratio (21.5%). Thus, the mean food neophobia value of Brazilian and Lebanese university students is lower than the Hungarian value; however, compared to the means of their own populations, both previous surveys indicate a level of neophobia higher than the Hungarian level. A sample of American university students [19] yielded different results; they had a significantly higher proportion of those open to novel food items (29.4%) and a lower proportion of food neophobia (13.4%) than Hungarian consumers, and the ratio of neutrals was also lower among them (57.2%).

The significant difference in Hungarian consumers’ food neophobia level compared to those of the consumers in other countries can be attributed to the different sample compositions in some cases being in line with previous findings [38]; although it also confirms the suggestion based on previous research results that the individual’s cultural background has a major influence on the person’s reaction to novel food items, that is, to the level of food neophobia (see, e.g., [4,28,39]). Our results, however, do not seem to verify findings by Choe and Cho [12] suggesting that Western cultures have a lower level of neophobia than Eastern cultures. The higher Hungarian food neophobia level revealed by our research is probably due to the fact that Hungarian (food) culture is more conservative and traditional [47,48], and consumers are more likely to reject novel food items in such a culture [4,25]. Besides this, we can also observe that the standard deviation of FNS values was somewhat higher in our research than in previous surveys, which indicates that although the Hungarian population can be regarded as strongly neophobic, food neophobia and food neophilia are both present among them, that is, the omnivore’s dilemma is very much at work here.

The high proportion of Hungarian consumers with a high level of food neophobia affects the development and marketing communication of food products intended for the Hungarian market. On the one hand, given that food neophobic consumers typically do not take part in the consumer testing of such items [5,19], the level of consumer acceptance indicated by such tests can seem higher than it actually is. In fact, according to Barrena and Sánchez [11], neophobia can be one of the reasons why the success rate of novel food items introduced to the market is so low. On the other hand, neophobic consumers may accept novel food items more easily if the manufacturer makes these items more familiar to the consumer by the use of well-known ingredients and flavors [12], and if this is emphasized in the marketing communication as well. Furthermore, such markets require more marketing efforts to combat neophobia, primarily with incentives that guarantee consumers’ high level of exposure to novel foods, such as product tasting, free samples or coupons [19].

In comparison with the results of previous research, we have found only a few—not consistent—similarities in terms of the items with which respondents can be regarded as mostly neophobic or mostly neophilic. Similarly to the case of Hungarian consumers, items 1 and 3 are among the items receiving the highest mean values in research conducted by Fernández-Ruiz et al. [4] and Tuorila et al. [17], item 1 is one of the items with the highest mean value in research conducted by Choe and Cho [12], as well as in that of Koivisto and Sjödén [16]. Similarly to the results of the present research, item 10 is the item with the highest mean value in the research of Olabi et al. [19] and Sogari et al. [25]. In the Hungarian sample, items 2 and 7 appeared among the lowest mean values, similarly to the results of Olabi et al. [19], Paupério et al. [28] and Sogari et al. [25]; item 7 has the lowest mean value in the research of Koivisto and Sjödén [16] as it does with Fernández-Ruiz et al. [4], while it is item 2 in Tuorila et al. [17]. Finally, as in the present research, item 9 has the lowest mean value in the study of Choe and Cho [12].

Similarly to previous research results [4,5,7,12,13,16,18,19,23,26,36], the level of food neophobia does not differ significantly between the genders among Hungarian consumers. Contrary to this, in some research studies [17,21,24], women proved to be less neophobic than men; Knaapila et al. [6] and Sogari et al. [25], however, arrived at a conclusion that women are more neophobic than men. FNS mean values increase with age among our respondents, just as in surveys made by D’Antuono and Bignami [18], Fernández-Ruiz et al. [4], Henriques et al. [5], Olabi et al. [19] and Tuorila et al. [17]. Paupério et al. [28] and Roseboom et al. [49], however, did not find a significant connection between age and the level of food neophobia, whereas Pliner and Melo [50] found a decrease in neophobia with age. According to some studies [17,19,27], urban consumers are less neophobic than rural ones; however, the present survey did not find a similar relationship. The literature [17,18,19,28] is consistent on the point that higher levels of education lead to lower levels of food neophobia, because education increases the chance of encountering different stimuli, therefore it increases the exposure to novel foods; this connection was verified by our research as well. Finally, contrary to previous findings [12,19] which did not indicate a connection between income and FNS values, in the case of Hungarian consumers, as their subjective income increases, their level of food neophobia decreases.

Factor analyses based on the FNS did not verify the previously assumed [36] one-dimensionality of the scale. Although some surveys did manage to identify one dimension for FNS, typically, they could only do this by leaving out certain items, such as Sogari et al. [25], who excluded item 8, Ritchey et al. [39], who left out items 2, 5, 8 and 9 (in US, Swedish and Finnish samples), and Guidetti et al. [1], who excluded items 3, 4, 8 and 9. However, most research—including the current study—revealed a two-factor solution (e.g., [4,12,17,19,28,39,51]), and in most of these—as in the results of the present survey—we can observe that the two factors clearly distinguish the negatively and positively worded (reversed) items. This, however, can be seen as some sort of a method effect, i.e., separation of items along the two factors is related to whether they are formulated in a direct or inverse way [51]. According to the results of a survey conducted by Olabi et al. [19], one factor is apprehension with regard to trying novel and ethnic foods, that is, it is related more to neophobic traits, and it contains the five negatively worded items, while the other factor is the interest in trying new foods, that is, it is related to more neophilic traits, and it contains the five positively worded (reversed) items. The same factor structure was found by Paupério et al. [28], Lenglet [51] and Ritchey et al. [39], with the difference that Paupério et al. [28] excluded item 8, Lenglet [51] left out items 8 and 9, while Ritchey et al. [39] excluded items 5 and 9 (in US and Swedish samples).

In the present research, the two-factor structure explored by Olabi et al. [19] was created by the exclusion of item 9 (“I will eat almost anything. (R)”). The problematic nature of this item has been pointed out by several studies; according to Ritchey et al. [39], for example, the item is too generic and misleading, because, for instance, vegetarians might be very willing to sample new foods, but they will not eat “anything”, since they do not eat meat; therefore, the item does not measure neophobia but something else. In a similar manner, Lenglet [51] and Zhao et al. [13] note that this item (along with item 8) indicates, instead, whether one is habitually a picky eater, and, according to Guidetti et al. [1], item 9 (along with items 3 and 8) does not differentiate between food neophobia and picky eaters, vegans/vegetarians and intolerant/allergic people. Related to this issue is the fact that although in research conducted by D’Antuono and Bignami [18] and by Zhao et al. [13], three factors were found, in both surveys one of the factors contained only items 8 and 9. The problem with item 9 is also indicated by the fact that its item-whole correlation was the lowest among all items in the original study of Pliner and Hobden [36]. Therefore Ritchey et al. [39] suggest that this item requires particular attention, and if it does not fit the scale, this is sufficient reason to exclude it from analysis.

In most of two-factor structures—as in the case of the present research—the factor containing the reversed items explains a higher ratio of variance than the factor containing the positively worded items (in the present research 47.927% vs. 17.325%), that is, the FNS measures neophilia more than it measures neophobia [51]. Accordingly, in the findings of Paupério et al. [28], the variance explained by the factor related to neophilia is 26.3%, that of the factor related to neophobia is 24.5%, whereas in Lenglet [51] the variance explained by the two factors is 49.7% and 13.2%, respectively. As opposed to this, in the research conducted by Olabi et al. [19], the variance explained by the two factors is 20% and 21.8%, respectively.

We managed to identify four clusters with the factors that emerged in the course of the analysis. Unlike our research, most other surveys—such as Bernal-Gil et al. [52] and Paupério et al. [28]—identified three clusters, but there were two-cluster solutions as well (e.g., [53]). Given that the different surveys used a different number of items in the FNS to serve as the basis of their analysis, and various numbers of factors with different compositions were created with different factor loadings, the possibilities of international comparisons of cluster analyses carried out with these factors is, therefore, severely limited.

## 5. Conclusions

When compared to international results, the level of food neophobia in Hungary is high, which should be taken into account by companies undertaking food innovations as both a distortion factor in market surveys and a possible barrier to the acceptance of food innovations. The reasons for this high level of food neophobia can ultimately be found in the conservative and traditional attitudes of Hungarian consumers, which are hard—almost impossible—to change. Despite the Hungarian population’s strong neophobic tendency, however, neophobia and neophilia are present at the same time. Of the groups established upon different background variables, the least neophobic are people aged 18–39, those who have finished secondary or higher education and those with a higher subjective income; this group can be a fruitful target of novel food products.

In the course of the exploratory factor analysis, we identified two distinct factors, which clearly separate items worded in a negative and positive way (reversed items). The first is the factor of “Willingness and trust,” in which the high factor loadings indicate that the dimension influences Hungarian consumers’ thinking to a great extent. The factor of “Rejection and pickiness” has less explanatory power, by which we proved that the FNS measures neophilia better than it measures neophobia.

The results of the cluster analysis can be useful in creating marketing strategies and policies, both for companies planning to launch novel food products or foods with unusual flavors, and for governmental or industry level organizations aiming to influence food consumption habits in a positive direction with the aim of improving the population’s health. Among adults surveyed, “Adventurous, open-minded” individuals constitute the ideal target group for the manufacturers of novel food items and products with unusual flavors, especially if those products also have health-enhancing and environment-friendly qualities; they are also a good target for marketing activities aiming to improve eating habits as well. They are innovators, early adopters and a portion of the early majority. They can be joined by “Careful and picky open-minded” individuals, who, similarly to the previous segment, can be receptive towards new food items, especially health-enhancing and environment-friendly ones, and restaurants offering new, unfamiliar flavors, particularly based on their demographic characteristics (mostly younger, more highly qualified people, singles, those in a better financial position and health- and environment-aware individuals). “Non-picky older adults”, on the other hand, constitute only a limited market for both the manufacturers of novel food items and restaurants offering unfamiliar flavors and governmental or industrial organizations’ marketing activities, partly because the size of this group is relatively small, but also due to its demographic features (older, less highly educated, people living on a tight budget and in smaller settlements). Finally, the group of “Traditional rejectors” cannot be considered an ideal target group, neither for the manufacturers of novel food items and restaurants offering new flavors, nor for health-enhancing marketing activities.

Our research can be regarded as unique for several reasons. First and foremost, to the best of the authors’ knowledge, this study was the first attempt to adapt and validate the FNS in Hungary. In the course of the research, we carried out not only a factor analysis based on FNS, but also a cluster analysis to identify consumer segments, which is also unique in Hungary. As a limitation, we have to mention that although the sample was representative of the Hungarian adult population, it would be beneficial to conduct the survey on a larger sample. The present research was conducted on an adult sample; in the future, however, it would be interesting to examine Hungarian children’s food neophobia level with the use of the Child FNS [54] in order to reveal the possible origins of the relatively high food neophobia level of Hungarian adults. In addition, further research will be necessary to find out whether Hungarian food neophobia level is similar to that in other Eastern European countries where the social and cultural backgrounds are very similar to those of Hungary.

## Figures and Tables

**Table 1:** Distribution of the sample according to the most important background variables (*n* = 500) and population composition according to representative variables.

Label	Sample Distribution		Population Distribution ^1^
	Count	%	%
Gender			
Male	236	47.2	47.8
Female	264	52.8	52.2
Age group			
18–39 years	164	32.8	33.2
40–59 years	175	35.0	34.7
60+ years	161	32.2	32.1
Settlement type			
Budapest	92	18.5	17.9
Other town	274	54.8	52.6
Village	134	26.7	29.5
Region			
Central Hungary	156	31.2	31.0
Transdanubia	151	30.2	29.9
Transtisza	193	38.6	39.1
Level of education			
Primary school	62	12.3	
Vocational school	142	28.4	
High school	206	41.1	
Higher education	91	18.1	
Subjective income			
Can live on it very well and can also save	38	7.7	
Can live on it but can save little	190	38.0	
Just enough to live on but cannot save	232	46.4	
Sometimes cannot make ends meet	24	4.8	
Have regular financial problems	1	0.2	
Not known/No answer	14	2.9	
Marital status			
Married	190	38.0	
Living with a live-in partner	97	19.4	
Widowed	60	12.0	
Single	98	19.6	
Divorced	53	10.5	
Separated from spouse	2	0.5	
Health awareness			
Not at all health-aware	20	3.9	
Mostly not health-aware	64	12.8	
Partly health-aware, partly not	193	38.7	
Mostly health-aware	167	33.5	
Very health-aware	47	9.4	
Not known/No answer	9	1.7	
Environment awareness			
Not at all environment-aware	9	1.8	
Mostly not environment-aware	43	8.5	
Partly environment-aware, partly not	146	29.3	
Mostly environment-aware	210	42.0	
Very environment-aware	84	16.7	
Not known/No answer	9	1.7	

^1^ Source of data: [40,42].

**Table 2:** Descriptive statistical indicators of the Food Neophobia Scale (FNS) (*n* = 500).

Statement ^1^	Statistical Indicator			
	Mean ^2^	Standard Deviation	Coefficient Variation (%)	Skewness
10. I like to try new ethnic restaurants. (R)	4.70	1.950	41.49	−0.328
3. If I don’t know what is in a food, I won’t try it.	4.52	1.934	42.79	−0.303
1. I am constantly sampling new and different foods. (R)	4.14	1.871	45.19	−0.038
6. At dinner parties, I will try a new food. (R)	4.08	1.901	46.59	0.098
5. Ethnic food looks weird to eat.	3.99	1.964	49.22	0.055
8. I am very particular about the foods I will eat.	3.94	1.906	48.37	−0.064
4. I like foods from different countries. (R)	3.93	1.882	47.89	0.156
7. I am afraid to eat things I have never had before.	3.68	1.911	51.93	0.106
2. I don’t trust new foods.	3.61	1.768	48.98	0.269
9. I will eat almost anything. (R)	3.18	1.788	56.23	0.567
**FNS total score**	**39.75**	**12.599**	**-**	**-**

^1^ In the case of items marked with (R), we reversed the values, that is, statements have to be understood in reverse. ^2^ A value of 1 stands for “strongly disagrees”, while a value of 7 stands for “strongly agrees”.

**Table 3:** Mean values of the FNS according to demographic variables.

	Mean	Standard Deviation	Test Value	*p* Value
*Gender*				
Male	40.37	12.466	t = 1.032	0.303
Female	39.18	12.718		
*Age group*				
18–39	36.87	11.550	F(2, 472) = 11.416	<0.001
40–59	39.02	12.973		
60+	43.47	12.368		
*Settlement type*				
Budapest	41.09	14.490	F(2, 472) = 0.657	0.519
Other town	39.53	12.083		
Village	39.24	12.213		
*Level of education*				
Primary school	45.19	13.280	F(3, 471) = 8.371	<0.001
Vocational school	42.25	12.380		
High school	37.19	12.078		
Higher education	38.67	12.173		
*Subjective income* ^1^				
Can live on it very well and can also save	36.20	11.959	F(3, 456) = 8.821	<0.001
Can live on it but can save little	36.74	11.568		
Just enough to live on but cannot save	41.65	12.570		
Sometimes cannot make ends meet	46.76	14.068		

^1^ Group “Have regular financial problems” was excluded, because only 1 respondent belongs to this group.

**Table 4:** Results of the exploratory factor analysis on the FNS.

Items	Willingness and Trust	Rejection and Particularity
10. I like to try new ethnic restaurants. (R)	0.836	
4. I like foods from different countries. (R)	0.830	
6. At dinner parties, I will try a new food. (R)	0.826	
1. I am constantly sampling new and different foods. (R)	0.776	
3. If I don’t know what is in a food, I won’t try it.		0.786
7. I am afraid to eat things I have never had before.		0.766
5. Ethnic food looks weird to eat.		0.732
8. I am very particular about the foods I will eat.		0.727
2. I don’t trust new foods.		0.600

Notes: extraction method: maximum likelihood; rotation method: varimax; rotation converged in 3 iterations; KMO = 0.865; Bartlett: (approx. chi sq.) 1912.429; (sig.) 0.000; communalities: 0.533–0.732; cumulative explained variance: 65.252; *n* = 500.

**Table 5:** The reliability of the measuring tools applied.

Factors	Cronbach’s Alpha	Composite Reliability	McDonald’s Omega
Willingness and trust	0.862	0.821	0.863
Rejection and particularity	0.822	0.883	0.827

Notes: *n* = 500; measurement scale: 1–7 interval scale.

**Table 6:** Results of confirmatory factor analysis.

Indicator	Acceptance Region ^1^	Empirical Results
CMIN/df	≥2 and ≤3	2.789
CFI	>0.9	0.978
GFI	>0.9	0.973
AGFI	>0.9	0.948
RMSEA	<0.07	0.062
SRMR	>0.9	0.962
NFI	>0.9	0.967
NNFI	>0.9	0.968

^1^ Source: [46].

**Table 7:** Results of examination of discriminant validity.

Factors	AVE	AVE Square Root	Correlation
Willingness and trust	0.668	0.817	0.476
Rejection and particularity	0.526	0.725	

**Table 8:** Distribution of clusters according to background variables (%) and mean FNS scores of clusters (*n* = 476).

Label	Cluster 1	Cluster 2	Cluster 3	Cluster 4
Size (%)	21.4	17.7	29.4	31.5
Gender ^1^				
Male	20.7	16.7	32.6	30.0
Female	21.8	18.5	26.6	33.1
Age group ^1^				
18–39 years ^2^	25.8	12.9	20.6	40.6
40–59 years ^2^	17.8	16.0	29.6	36.7
60+ years	20.4	25.0	38.8	15.8
Settlement type ^1^				
Budapest ^2^	15.4	14.3	40.7	29.7
Other town	28.3	14.2	26.0	31.5
Village	12.3	26.9	28.5	32.3
Region ^1^				
Central Hungary ^2^	19.7	15.1	28.9	36.2
Transdanubia	21.6	15.5	33.8	29.1
Transtisza	22.7	22.2	25.6	29.5
Level of education ^1^				
Primary school	13.2	28.3	37.7	20.8
Vocational school	18.8	24.8	36.1	20.3
High school ^2^	22.5	13.2	24.5	39.7
Higher education ^2^	26.7	11.6	25.6	36.0
Subjective income ^1^				
Can live on it very well and can also save ^2^	27.8	16.7	25.0	30.6
Can live on it but can save little	29.4	17.2	17.8	35.6
Just enough to live on but cannot save ^2^	16.7	18.9	35.5	28.8
Sometimes cannot make ends meet	0.0	17.4	52.2	30.4
Have regular financial problems	0.0	100.0	0.0	0.0
Not known/No answer ^2^	7.1	7.1	57.1	28.6
Marital status ^1^				
Married	20.0	17.2	31.1	31.7
Living with a live-in partner ^2^	20.9	14.3	24.2	40.7
Widowed ^2^	16.9	30.5	32.2	20.3
Single ^2^	27.7	16.0	27.7	28.7
Divorced ^2^	18.4	16.3	32.7	32.7
Separated from spouse	50.0	0.0	50.0	0.0
Health awareness ^1^				
Not at all health-aware	15.0	15.0	40.0	30.0
Mostly not health-aware	23.3	15.0	30.0	31.7
Partly health-aware, partly not	20.5	22.2	31.4	25.9
Mostly health-aware ^1^	16.6	15.3	26.8	41.4
Very health-aware ^1^	43.2	15.9	20.5	20.5
Not known/No answer	0.0	11.1	55.6	33.3
Environment awareness ^1^				
Not at all environment-aware ^2^	22.2	11.1	33.3	33.3
Mostly not environment-aware	22.0	17.1	34.1	26.8
Partly environment-aware, partly not	18.0	18.0	34.5	29.5
Mostly environment-aware	19.1	16.6	27.6	36.7
Very environment-aware ^1^	34.2	22.8	19.0	24.1
Not known/No answer	0.0	11.1	55.6	33.3
FNS total score	38.65	37.68	54.18	28.17
FNS items				
1. I am constantly sampling new and different foods. (R)	3.07	4.99	5.70	2.94
2. I don’t trust new foods.	3.78	2.94	5.12	2.51
3. If I don’t know what is in a food, I won’t try it.	5.58	2.85	6.02	3.40
4. I like foods from different countries. (R)	2.78	4.69	5.62	2.65
5. Ethnic food looks weird to eat.	4.58	2.99	5.58	2.69
6. At dinner parties, I will try a new food. (R)	3.01	5.12	5.62	2.78
7. I am afraid to eat things I have never had before.	4.41	2.44	5.28	2.39
8. I am very particular about the foods I will eat.	5.17	2.45	4.88	3.06
9. I will eat almost anything. (R)	2.96	3.16	4.11	2.42
10. I like to try new ethnic restaurants. (R)	3.31	6.05	6.25	3.33

^1^ % within background variables (row%). ^2^ The 0.1% difference in the sum of the percentage distributions of the clusters from 100% derives from rounding.

## Data Availability

The data presented in this study are available on request from the corresponding author.

## References

[1] Guidetti M., Carraro L., Cavazza N., Roccato M. (2018). Validation of the revised Food Neophobia Scale (FNS-R) in the Italian context. Appetite.

[2] Rozin P., Apt C.M. (1977). The use of characteristic flavorings in human culinary practice. Flavor: Its Chemical, Behavioral, and Commercial Aspects. Proceedings of the Arthur D. Little Flavor Symposium.

[3] Raudenbush B., Frank R.A. (1999). Assessing food neophobia: The role of stimulus familiarity. Appetite.

[4] Fernández-Ruiz V., Claret A., Chaya C. (2013). Testing a Spanish-version of the Food Neophobia Scale. Food Qual. Prefer..

[5] Henriques A.S., King S.C., Meiselman H.L. (2009). Consumer segmentation based on food neophobia and its application to product development. Food Qual. Prefer..

[6] Knaapila A., Silventoinen K., Broms U., Rose R.J., Perola M., Kaprio J., Tuorila H.M. (2011). Food neophobia in young adults. Genetic architecture and relation to personality, pleasantness and use frequency of foods, and body mass index—A twin study. Behav. Genet..

[7] Falciglia G.A., Couch S.C., Gribble L.S., Pabst S.M., Frank R. (2000). Food neophobia in childhood affects dietary variety. J. Am. Diet Assoc..

[8] Rubio B., Rigal N., Boireau-Ducept N., Mallet P., Meyer T. (2008). Measuring willingness to try new foods: A self-report questionnaire for French-speaking children. Appetite.

[9] Alley T.R., Potter K.A., Preedy V., Watson R., Martin C. (2011). Food Neophobia and Sensation Seeking. Handbook of Behavior, Food and Nutrition.

[10] Regulation (EU) 2015/2283 of the European Parliament and of the Council of 25 November 2015 on Novel Foods. https://data.europa.eu/eli/reg/2015/2283/oj.

[11] Barrena R., Sánchez M. (2013). Neophobia, personal consumer values and novel food acceptance. Food Qual. Prefer..

[12] Choe J.Y., Cho M.S. (2011). Food neophobia and willingness to try non-traditional foods for Koreans. Food Qual. Prefer..

[13] Zhao J., Gao Z., Li Y., Wang Y., Zhang X., Zou L. (2020). The Food Neophobia Scale (FNS): Exploration and confirmation of factor structure in a healthy Chinese sample. Food Qual. Prefer..

[14] Bánáti D., Lakner Z. (2006). Knowledge and Acceptance of Genetically Modified Foodstuffs in Hungary. J. Food Nutr. Res..

[15] Bánáti D., Mészáros Z., Szabó E. (2018). Attitude toward the cloning of animals for food in Hungary (Állatok élelmezési célú klónozásának megítélése Magyarországon). J. Food Investig..

[16] Koivisto U.-K., Sjödén P.-O. (1996). Food and general neophobia in Swedish families: Parent-child comparisons and relationships with serving specific foods. Appetite.

[17] Tuorila H., La L., Pohjalainen L., Lotti L. (2001). Food neophobia among the Finns and related responses to familiar and unfamiliar foods. Food Qual. Prefer..

[18] D’Antuono L.F., Bignami C. (2012). Perception of typical Ukrainian foods among an Italian population. Food Qual. Prefer..

[19] Olabi A., Najm N.E.O., Baghdadi O.K., Morton J.M. (2009). Food neophobia levels of Lebanese and American college students. Food Qual. Prefer..

[20] Bäckström A., Prittila-Backman A.-M., Tuorila H. (2004). Willingness to try new foods as predicted by social representations and attitude and trait scales. Appetite.

[21] Siegrist M., Hartmann C., Keller C. (2013). Antecedents of food neophobia and its association with eating behavior and food choices. Food Qual Prefer.

[22] Dovey T.M., Staples P.A., Gibson E.L., Halford J.C.G. (2008). Food neophobia and “picky/fussy” eating in children: A review. Appetite.

[23] Rossbach S., Foterek K., Schmidt I., Hilbig A., Alexy U. (2016). Food neophobia in German adolescents: Determinants and association with dietary habits. Appetite.

[24] Koivisto Hursti U.-K., Sjödén P.-O. (1997). Food and general neophobia and their relationship with self-reported food choice: Familial resemblance in Swedish families with children of ages 7 ± 17 years. Appetite.

[25] Sogari G., Menozzi D., Mora C. (2019). The Food Neophobia Scale and young adults’ intention to eat insect products. Int. J. Consum. Stud..

[26] Cooke L., Wardle J., Gibson E.J. (2003). Relationship between parental report of food neophobia and everyday food consumption in 2–6-year-old children. Appetite.

[27] Flight I., Leppard P., Cox D.N. (2003). Food neophobia and associations with cultural diversity and socio-economic status amongst rural and urban Australian adolescents. Appetite.

[28] Paupério A., Severo M., Lopes C., Moreira P., Cooke L., Oliveira A. (2014). Could the Food Neophobia Scale be adapted to pregnant women? A confirmatory factor analysis in a Portuguese sample. Appetite.

[29] Ribeiro de Andrade Previato H.D., Behrens J.H. (2015). Translation and validation of the Food Neophobia Scale (FNS) to the Brazilian Portuguese. Nutr. Hosp..

[30] Addessi E., Galloway A.T., Visalberghi E., Birch L.L. (2005). Specific social influences on the acceptance of novel foods in 2–5-year-old children. Appetite.

[31] Lafraire J., Rioux C., Giboreau A., Picard D. (2016). Food rejections in children: Cognitive and social/environmental factors involved in food neophobia and picky/fussy eating behaviour. Appetite.

[32] Capiola A., Raudenbush B. (2012). The effects of food neophobia and food neophilia on diet and metabolic processing. Food Nutr. Sci..

[33] Galloway A.T., Lee Y., Birch L.L. (2003). Predictors and consequences of Food neophobia and pickiness in young girls. J. Am. Diet Assoc..

[34] Kaar J.L., Shapiro A.L.B., Fell D.M., Johnson S.L. (2016). Parental feeding practices, food neophobia, and child food preferences: What combination of factors results in children eating a variety of foods?. Food Qual. Prefer..

[35] Birch L.L., McPhee L., Shoba B.C., Pirok E., Steinberg L. (1987). What kind of exposure reduces children’s food neophobia? Looking vs. tasting. Appetite.

[36] Pliner P., Hobden K. (1992). Development of a scale to measure the trait of food neophobia in humans. Appetite.

[37] Schickenberg B., Van Assema P., Brug J., De Vries N.K. (2008). Are the Dutch acquainted with and willing to try healthful food products? The role of food neophobia. Public Health Nutr..

[38] Rabadán A., Bernabéu R. (2021). A systematic review of studies using the Food Neophobia Scale: Conclusions from thirty years of studies. Food Qual. Prefer..

[39] Ritchey P.N., Frank R.A., Koivisto Hursti U.-K., Tuorila H. (2003). Validation and cross-national comparison of the Food Neophobia Scale (FNS) using confirmatory factor analysis. Appetite.

[40] Hungarian Central Statistical Office Tables (STADAT)-Time Series of Annual Data-Population, vital events/1.2.Population by Type of Settlement, 1 January (1980–2019), 1.3.Population by Sex and Age, 1 January (1980–2019). http://www.ksh.hu/stadat_annual_1.

[41] Gill J., Johnson P. (2010). Research Methods for Managers.

[42] Hungarian Central Statistical Office Tables (STADAT)-Time Series of Annual, Regional Statistics-Population, Vital events/6.1.1.Resident Population by Sex, 1 January (2001–2019). http://www.ksh.hu/stadat_annual_6_1.

[43] Elkins A., Zickgraf H.F. (2018). Picky eating and food neophobia: Resemblance and agreement in parent/young adult dyads. Appetite.

[44] Fornell C., Larcker D.F. (1981). Evaluating structural equation models with unobservable variables and measurement error. J. Mark Res..

[45] Cronbach L.J. (1951). Coefficient alpha and the internal structure of tests. Psychometrika.

[46] Hooper D., Coughlan J., Mullen M. (2008). Structural Equation Modelling: Guidelines for Determining Model Fit. Electron. J. Bus Res. Methods.

[47] Bánáti D. (2020). Veggie burgers, vegan meats? The ruling of the European Parliament paved the way for meat substitutes with meat denominations (Vega hamburgerek, vegán húsok? Az Európai Parlament döntése a növényi alapú húspótló élelmiszerek elnevezéséről). J. Food Investig..

[48] Kovács Á., Zsarnóczay G. (2007). Protected meat products in Hungary–local foods and hungaricums. Anthropol. J. Food.

[49] Roseboom T., de Rooij S., Painter R. (2006). The Dutch famine and its long-term consequences for adult health. Early Hum. Dev..

[50] Pliner P., Melo N. (1997). Food neophobia in humans: Effects of manipulated arousal and individual differences in sensation seeking. Physiol. Behav..

[51] Lenglet F. (2018). FNS or the Varseek-scale?: Proposals for a valid operationalization of neophilia. Food Qual. Prefer..

[52] Bernal-Gil N.Y., Favila-Cisneros H.J., Zaragoza-Alonso J., Cuffia F., Rojas-Rivas E. (2020). Using projective techniques and Food Neophobia Scale to explore the perception of traditional ethnic foods in Central Mexico: A preliminary study on the beverage Sende. J. Sens. Stud..

[53] Johns N., Edwards J.S.A., Hartwell H. (2011). Food neophobia and the adoption of new food products. Nutr. Food Sci..

[54] Pliner P. (1994). Development of Measures of Food Neophobia in Children. Appetite.

